# Laughter, play faces and mimicry in animals: evolution and social functions

**DOI:** 10.1098/rstb.2021.0177

**Published:** 2022-11-07

**Authors:** Marina Davila-Ross, Elisabetta Palagi

**Affiliations:** ^1^ Psychology Department, King Henry Building, University of Portsmouth, Portsmouth PO1 2DY, UK; ^2^ Department of Biology, Ethology Unit, University of Pisa, Via A. Volta 6, 56126, Pisa, Italy

**Keywords:** laughter, laugh faces, play expressions, animal play, mimicry, evolution

## Abstract

Human laughter and laugh faces show similarities in morphology and function with animal playful expressions. To better understand primordial uses and effects of human laughter and laugh faces, it is important to examine these positive expressions in animals from both homologous and analogous systems. Phylogenetic research on hominids provided empirical evidence on shared ancestry across these emotional expressions, including human laughter and laugh faces. In addition, playful expressions of animals, in general, arguably have a key role in the development of social cognitive skills, a role that may help explain their polyphyletic history. The present work examines the evolution and function of playful expressions in primates and other animals. As part of this effort, we also coded for muscle activations of six carnivore taxa with regard to their open-mouth faces of play; our findings provide evidence that these carnivore expressions are homologues of primate open-mouth faces of play. Furthermore, our work discusses how the expressions of animal play may communicate positive emotions to conspecifics and how the motor resonance of these expressions increases affiliation and bonding between the subjects, resembling in a number of ways the important social–emotional effects that laughter and laugh faces have in humans.

This article is part of the theme issue ‘Cracking the laugh code: laughter through the lens of biology, psychology and neuroscience’.

## 1. Introduction

Laugh vocalizations (from here on ‘laughter’) and laugh faces occupy a central role in human social cohesion, occurring in a wide range of daily interactions with friends and strangers [1–3]. They promote the development of cognitive and socio-emotional skills [4,5] and may affect health and well-being [6–8]. Early along the ontogenetic trajectory, they frequently take place within the context of play, where laughter and laugh faces of children show notable commonalities in form and function with play expressions of nonhuman animals [9–11].

In the current work, we review the literature on vocal and facial expressions of play in nonhuman animals. We examine evolutionary models of laughter and laugh faces, with special focus on great apes, and moving on to expressions of play in other animals, with a discussion on both homologous and analogous behaviours. Furthermore, we examine the occurrence and function of these expressions, with a special focus on mimicry. This review will lean on empirical studies on animal playful behaviours, many of which have been published within the last couple of decades with advanced technologies in behavioural coding, and they will be discussed in line with theoretical works.

## 2. Evolution of vocal and facial expressions of play

### (a) Laughter and laugh faces in hominids

Humans and nonhuman animals show interesting commonalities in both anatomy and context in the vocal and facial expressions of play, expressions that occur early along the developmental trajectory [12–15]. Interestingly, great apes produce play vocalizations that show similarities with human laughter [10,11]. Often, these vocalizations involve a series of low-frequency staccato grunts that can be easily induced by tickling in infants and juveniles [16,17]. These vocalizations predominantly accompany open-mouth faces (play faces), facial expressions of play that often occur on their own, i.e. as silent expressions [18–20].

Such similarities in morphology and occurrence may naturally lead to the notion of evolutionary continuity from primordial play expressions to human laughter and laugh faces of positive affect. In order to systematically reconstruct the evolutionary pathways of human hard-wired behaviours, it is key to lean on the principle of maximum-parsimony, where the most likely explanation involves the least number of predicted evolutionary changes [21]. Thus, the first step toward reconstructing laughter and laugh face evolution is to examine hominid expressions within the context of play, thereby making predictions about the last common ancestral ape species of extant great apes and modern humans.

To place laugh vocalizations within the multiplex phylogeny of the Hominidae, Davila Ross *et al*. [22] analysed tickling-induced vocalizations of immature great apes and human infants. Their phylogenetic trees based on acoustic data revealed a topology identical to the well-established hominid tree generated by a series of genetic studies (e.g. [23–25]), suggesting shared ancestry of the examined vocalizations, which included human infant laughter [22]. Thus, evidence for laughter in great apes was provided as well as a foundation that is based on the principle of maximum-parsimony for phylogenetically testing the relationship between great ape open-mouth faces and human laugh faces.

Building on the laughter research, Davila-Ross *et al*. [26] examined the facial muscle movements in laughing chimpanzees via ChimpFACS [27]. These chimpanzees were found to part their lips while dropping/stretching their jaws and often they would also pull both lip corners back and upwards and raise their upper lips (revealing their upper teeth) as well as their cheeks (causing wrinkles around the eyes, i.e. crow's feet) [26]. These facial movements of the apes matched those of laughing humans that were measured with FACS [28–32]. Collectively, the two studies revealed that human laughter as well as laugh faces have a pre-human basis (for a more detailed reconstruction, see [33]).

**Figure 1.**
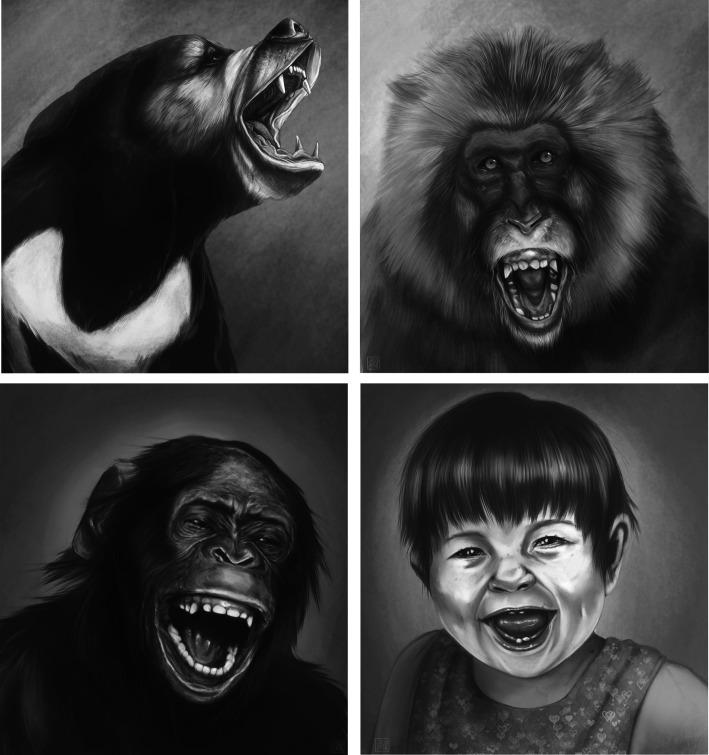
Illustrations of a sun bear, a Japanese macaque, a bonobo and a human child producing playful facial expressions with upper-teeth exposure. The artist (Fosca Mastrandrea) created these drawings *ex novo*; for accuracy, she leaned on photographs that were available for each species.

The finding of the upper-teeth exposure in laughing chimpanzees [26] was in line with observations of silent open-mouth faces in playing great apes [34–38]. For illustrations of the upper-teeth exposure, which is primarily caused by the contraction of the levator labii superioris muscle, in four species during play, see figure 1. These displays (also referred to as ‘relaxed open-mouth bared-teeth displays' or ‘full play faces’) stand in contrast to the ‘relaxed open-mouth display’, which shows a relaxed upper lip with the upper teeth covered (also see [39,40]). In his pioneering work on the evolution of smiles, van Hooff [41] found that the chimpanzees at Burgers' Zoo did not activate this facial muscle while laughing and playing. Thus, the open-mouth faces were not considered in his work to be phylogenetically continuous from nonhuman to human expressions [41].

Consequently, the most parsimonious explanation for the evolution of human laughter and laugh faces of positive affect, based on acoustic data and facial muscle activations, is that they evolved directly within the context of play in ancestral nonhuman species. This explanation involves no major evolutionary changes within hominid phylogeny. Consistent with this notion, the Complexity and Continuity Hypothesis was recently introduced to provide an evolutionary reconstruction of hominid laughter and laugh faces, primarily leaning on empirical findings on hominid play expressions and their variants over the last two decades [33]. According to this hypothesis, both human laughter and laugh faces of positive affect most likely evolved within the context of play in pre-human times and were already complex in both form and function when produced by ancestral species.

### (b) Reconstructing beyond the hominids

The open-mouth face found in great ape play is likely to have deep roots in mammal biology. Expressions of wide open-mouths during play are also common in other primates as well as in carnivores [42–45]. Besides primates and carnivores, horses have been observed to produce open-mouth faces of play [46]. Although the facial physiognomy of primates and carnivores differs notably, the underlying muscles responsible for such wide open-mouth movements during play are comparable across these taxonomic groups ([26,47–49]; also figure 1). Whereas other types of facial movements have additionally been reported for play, e.g. ear movements [50,51] and puckered lips [52], it seems reasonable to consider that the open-mouth faces of primates and carnivores are homologues since evolution does not tend to eradicate and rebuild comparable biological systems.

To gain further insight into this topic, we examined the action units (AUs) of the open-mouth faces of six carnivores, i.e. three Caniformia taxa (Czechoslovakian wolfdogs, *Canis lupus familiaris*; Bornean sun bears, *Helarctos malayanus euryspilus*; African wild dogs, *Lycaon pictus*) and three Feliformia taxa (spotted hyaenas, *Crocuta crocuta*; meerkats, *Suricata suricatta*; lions, *Panthera leo*). We tested for the presence of muscle activations in open-mouth faces of carnivore play, based on comparable activations that were previously found in primate play [26,47]. Our analysis revealed that the six predominant AUs found in open-mouth faces of primate play (Upper Lip Raiser, Lip Corner Puller, Lower Lip Depressor, Lips Parted, Jaw Drop and Mouth Stretch) show comparable activations in open-mouth faces of both caniform and feliform play (table 1). The prototypical open-mouth face of the carnivores seems to consist of AU25 (Lips Parted) (orbicularis oris, caninus, levator labii maxillaris, levator nasolabialis, platysma) and the mutually exclusive nonmimetic muscles AU26/AU27 (Jaw Drop/Mouth Stretch), but there may also be different combinations of AUs, such as the presence of AU12 (Lip Corner Puller) (zygomaticus major), which is also found in primates. Consequently, our findings provide evidence that the open-mouth faces of play in carnivores and primates are homologues. It needs to be noted, however, that carnivores show some differences in their facial musculature when compared with primates, for instance, in the caniform platysma, compared with the primate depressor labii inferioris (for a review, see [53]).

**Table 1.** Overview of muscle activations found in six Carnivora taxa. Caniformia: Czechoslovakian wolfdogs, Bornean sun bears and African wild dogs. Feliformia: spotted hyaenas, meerkats^a^ and lions^a^. Fifty-nine open-mouth faces were coded from Czechoslovakian wolfdogs (Italy), 20 from rehabilitant sun bears at the Bornean Sun Bear Conservation Centre (Malaysia), 10 from wild dogs at the Dvur Kralove Zoo (Czech Republic), and 10 from wild spotted hyaenas at the Siyafunda Wildlife & Conservation (Limpopo, South Africa).

action unit (AU)	wolfdog	sun bear	wild dog	hyaena	meerkat	lion
AU109 + 110 Nose Wrinkler + Upper Lip Raiser	present	present	present	present	present	present
AU12 Lip Corner Puller	present	present	present	present	present	present
AU16/116 Lower Lip Depressor	not coded	present	not found	not found	present	present
AU25 Lips Parted	present	present	present	present	present	present
AU26 Jaw Drop	present	present	present	present	present	present
AU27 Mouth Stretch	present	present	present	present	present	present

^a^Online videos coded on 6 February 2022: https://www.smithsonianmag.com/videos/category/smithsonian-channel/baby-meerkats-at-play/; https://www.youtube.com/watch?v=KYM57FroGQ8. https://www.youtube.com/watch?v=PjjIQvjZ1Qc; https://www.youtube.com/watch?v=TeCkm-BEZ-8.

Across species, animals seem to display multiple open-mouth face variants and their predominant use may vary [36,40,54,55]. Such variation is based on a number of additional muscle activations that add to the facial complexity of the wide open-mouths, such as the exposure of the upper teeth and/or the pulling back of the lip corners [54,56–58]. Whereas species-specific differences may be explained by multiple factors, one explanation has received notable attention in research.

The Power Asymmetry Hypothesis proposed by Preuschoft & van Hooff [59] provides an interesting explanation for different occurrences of the upper-teeth exposure during play and the lack thereof. According to this hypothesis, animals of steeper hierarchies are likely to produce signals of play that are more easily distinguishable from other expressions with wide-open mouths than animals of more relaxed social systems. Such clear signalling is likely to reduce the chances of miscommunication and, consequently, the chances of rough play escalating into fights. By contrast, species of more relaxed social systems would not need to provide such distinctive signals. Empirical evidence based on taxonomic group comparisons support this hypothesis for primates. For instance, pig-tailed macaques and chimpanzees expose less often their upper teeth during play than Tonkean macaques and bonobos [39,40,60]. This way, the open-mouth faces of play are more distinct from other open-mouth faces in the former primates, who live in general within steeper hierarchies than the latter. It would be interesting to test the hypothesis for social carnivores.

The physical properties of the open-mouth face might provide us further insight into its origin. Interestingly, both primates and carnivores show facial movements when producing open-mouth faces during play that are comparable with those of play biting [45,61,62]. Early on, such commonality led to the prediction that this play expression may have evolved from play biting through a ritualization process [63–65]. At an evolutionary level, the exaggeration and formalization of specific motor actions forming a functional pattern (e.g. play bite) can emerge into a new behaviour (e.g. open-mouth face) specifically designed to communicate [66]. In rough types of play, such as play fighting, it may be essential to show such an exaggerated signal in order to convey the message ‘this is play’ and avoid escalation into real fighting.

It is likely that open-mouth faces existed already prior to the origin of laugh vocalizations. Open-mouth faces are often produced by themselves and, consequently, independent of laughter as well as other play vocalizations. By contrast, laughter of hominids is often accompanied by open-mouth faces [56]. Furthermore, open-mouth faces develop earlier than laughter and other play vocalizations in nonhuman primates and humans [13,67–69] and as these expressions are hard-wired; this pattern arguably fits within Ernst Haeckel's [70] recapitulation theory, where ‘ontogeny recapitulates phylogeny’ for morphological traits.

Play vocalizations are also produced among unrelated mammalian taxa, such as rodents, canids, elephants and dolphins (see [71–74]). These vocalizations occur even beyond the placental mammal classification, including marsupials and parrots [74,75], where the vocal production system is clearly analogous. Some of these vocalizations may, therefore, not be related, while others might be—it is difficult to arrive at conclusions here without phylogenetic analyses. Interestingly, primates and carnivores sometimes produce panting play vocalizations and such acoustic feature suggests that these vocalizations evolved from heavy breathing in rough-and-tumble play, perhaps even based on the same origin [11,74].

Within the primate order, not all play vocalizations can represent laughter because a number of species produce more than one type of play vocalization. For instance, orangutans produce high-pitch play squeaks in addition to their low-pitch grunt-like laughter during play and when tickled that are distinct in sound production [76]. The former is produced by regular vocal-fold vibrations and the latter is a call of deterministic chaos. Some gibbon species also produce play squeaks (*Nomascus* spp.: T. Geissmann 2007, personal communication), while others produce play vocalizations that more resemble orangutan laughter (e.g. *Symphalangus syndactylus*: 76; *Hylobates lar*: E. Zimmermann 2007, personal observation). Similarly, children vocalize not only laughter as nonverbal expressions of play; for instance, they may also produce squeals as positive expressions [77]. The mere presence of acoustically distinct types of play vocalizations within a species indicates that play vocalizations, in general, are the result of a polyphyletic history, where at least two of these vocalization types have different origins.

While speculations on play vocalization phylogeny are interesting, it may be difficult without phylogenetic analyses to draw conclusions about how these vocalizations relate beyond hominids. However, the case for ‘laughter’ weakens for taxonomic groups where play vocalizations do not seem to accompany open-mouth faces, such as rats. Finlayson and colleagues [50] specifically examined the facial movements of playful rats during tickling. Whereas the rats showed positive facial behaviours, such as the moving of the ears, the researchers never observed an opening of the mouth during play [50], findings that are consistent with other observations (S. Pellis and M. Schweinfurth 2007, personal communication). Nonetheless, the ultrasonic play vocalizations of rats clearly reflect a positive state and have important social functions, with the rats following the human hand that tickles them [78,79]. As a result, play vocalizations cannot *a priori* be considered to share ancestry with laugh vocalizations. The more distanced animals are from the hominid clade, the higher the chances are that their play vocalizations have a different evolutionary root from laughter.

## 3. Social use of open-mouth faces, laughter and other play vocalizations

### (a) Play coordination, social cohesion and the development of skills

The differences in modality and anatomy of play expressions as well as their polyphyletic history indicate that these expressions have a complexity in both form and function. Carnivores and primates seem to modify their play expressions when they receive the attention of their playmates [38,45,58,80,81], and chimpanzees and bonobos are known to also modify them if the mothers of their infant playmates are nearby [82] or group members are attentive to the sender [83,84]. Multiple social functions of play expressions that are not necessarily mutually exclusive have been discussed.

As mentioned earlier in the present work, an important function of animal play expressions is to signal ‘this is play’, which helps to coordinate actions among playmates [46,85–87]. Probably most importantly, such signalling is likely to help avoid escalation into real fights during rougher play and, consequently, to prevent getting hurt, especially when the playmates are dissimilar in strength and do not have close social relationships [74,88–90].

Whereas mammals produce both types of open-mouth faces in both gentle and rough play [35,58], their upper-teeth exposure, which resembles wide-open mouth displays of submission and appeasement [39,54], tends to occur more often during rough play [37,60,91]. Similarly, play vocalizations seem to be predominantly produced in rough-and-tumble [74]. Thus, these types of expressions might signal to the recipient ‘this is just play’. It is also possible that the playmates widen their mouths further and expose their teeth owing to having to breathe more intensely and loudly during rough-and-tumble. Furthermore, the individuals producing these expressions could be in a state of high arousal and show more play biting [56]. The open-mouth faces without exposed teeth, on the other hand, seem to be less dependent on play intensity and have a more general application within play [37,56,60].

Consistent with the claim that play expressions signal ‘this is play’ or ‘this is just play’, empirical findings show that these expressions from rodents to primates may permit play actions and play bouts to be prolonged [58,84,86,87,89,92,93]. Furthermore, animals can sometimes produce open-mouth faces as part of the play invitation (e.g. when hitting the other playmate prior to play), and such signalling here is likely to help invite to play [60]. Such increase in playful interactions, key affiliative behaviours in social animals, is likely to have a notable impact on social bonding and, consequently, other behaviours among group members [43,94–98]. In humans, it is also known that laughter helps social cohesion [1,99]. Five-month-old infants already respond differently when hearing friends laughing together compared with strangers behaving this way [100].

In accordance with Barbara Fredrickson's Broaden-and-Build Theory of Positive Emotions, play expressions may contribute to the development of a range of skills that are central for individuals living in social groups, including social-cognitive skills [4]. Supporting this claim, play may consist of cooperative and competitive behaviours, where young individuals can practise with low risk a range of behaviours and further explore the impact these behaviours have on their conspecifics [93,101], which may become more relevant at a later stage in their development [102]. Such functions are consistent with the notion that positive expressions, such as laughter and other play expressions, do not necessarily need to constantly have immediate benefits, and perhaps their range in function makes them different from negative expressions, where it can be crucial to respond quickly in a risky situation [4,5].

Despite overlapping contexts, play vocalizations and open-mouth faces are at least to some extent likely to differ in function. Play vocalizations seem to be more limited to the context of play than open-mouth faces ([84]; for functional flexibility, see [103,104]). Although open-mouth faces occur predominantly during play bouts, they have also been observed shortly prior to them in order to invite a conspecific to play [105]. On a few occasions, they have been observed fully outside of play. One such incident took place at the Serengeti Park Hodenhagen, where Pia, a juvenile chimpanzee, was unsuccessfully play-inviting her father by pulling his hair (see [33]). As he did not budge, Pia left the scene, laid down to relax for a while, and suddenly started producing open-mouth faces (for a video footage, see [33]). Such rare incidents, where open-mouth faces that occur after nonaggressive violations of expectations resemble the use of human laughter linked to benign violations and humour [106,107], can already be observed in humans during infancy [108].

Perhaps the main difference in playful expressions between human and nonhuman animals lies in their occurrence. Human laughter and laugh faces with their sophisticated volitional as well as spontaneous forms are characteristic components of human everyday social interactions that may certainly vary in function and express, for instance, politeness, embarrassment, mocking and Schadenfreude [9,11,109,110]. They show a level of control that has, to our knowledge, not been found for animal play expressions, at least thus far.

### (b) Mimicking and why it may be important for animals

The matching of expressions has a special role in animal play, where the expression of one playmate induces the same expression in another playmate. It has been mainly studied in the form of mimicry (e.g. dogs–horses [46], carnivores [58,62,111,112], primates [26,34,86,92,113]). Mimicry involves an automatic response system that is perhaps most easily observable as rapid mimicry, with a response latency of 1 s or less [114,115]. Rapid mimicry within short-distance communication has been predominantly observed in playful contexts, perhaps because they represent a platform for acquiring a range of social, emotional and cognitive skills [4,5].

The matching of animal play expressions, however, also comes in other types. For instance, delayed matching responses have been reported for primate open-mouth faces and play vocalizations [86,92,112]. Although caution is necessary when discussing why these responses were slower than rapid mimicry, it is interesting to note that humans sometimes respond more slowly when the behaviour is volitional, because additional neural processes are then involved compared with rapid mimicry (see [114,116,117]). Furthermore, the matching of play expressions among animals may range from being exact, i.e. with the same variant matched (e.g. open-mouth faces with upper lips raised [58,112]), to being distinct, i.e. with a different variant of the same expression matched (e.g. long laugh bouts seem to induce short laugh bouts [86]). Interestingly, previous studies have examined only dyadic constellations, so that research is needed to quantitatively explore if triadic facial expressions can occur in primates and other animals.

Thus, the matching of play expressions comes in various types, suggesting that they take up important functions among animals. Such matching is likely to heightened advantages that already come with spontaneously producing play expressions. Owing to its facial and vocal feedback component, it may serve even further as a social glue than spontaneous play expressions and may also contribute more to modulating interactions among playing animals [58,92]. In lowland gorillas, for instance, Bresciani and colleagues [118] found that such matching is prolonged when the facial response of the receiver mirrors the facial constellation of the playmate.

Although it can be problematic to link behavioural actions consistently with emotional states [119–121], expressions of play seem to be, in general, closely associated with positive affect in both nonhuman animals and humans (see [33]). Perhaps the context of gentle solitary play shows its link to positive states in animals most readily. For example, expressions produced by a young animal playing by her/himself are unlikely to have an interactive application value, making it reasonable to argue that such expressions are positive emotional outbursts. Such a link to affect may certainly be sustained during social interactions. Consequently, the mirror effect of play expressions may well be linked to elevated valence arousal states among playmates.

Two distinctive pathways that may lead to such an elevated state have received notable research attention [122–125]. First, the matching response is induced on a motoric level, a pathway that has been discussed in relation with behavioural contagion [126,127] as well as motor mimicry [122,128]. In this case, a spontaneous play expression triggers the same expression in the other playmate. Especially for motor mimicry, it has been argued that the motor resonance may trigger in the recipient the same emotional state experienced by the playmate [34,128,129]. However, emotions do not necessarily need to be involved when a behaviour is matched. The matching of behaviours may indicate, for instance, that the playmates are already in comparable states, perhaps in elevated positive states, which could help to prolong play. Interestingly, studies on animal play have shown that rapid facial mimicry and delayed laugh responses are linked to longer play bouts [34,58,62,86,92]. Whereas not all matching expressions in play must be linked to affect, it seems reasonable to argue that this association will strengthen over time, especially since young animals typically experience myriad playful events with familiar conspecifics.

Second, the matching response is affect-induced [122,130]. Here, a spontaneous play expression of a playmate causes an elevated positive state in the other playmate, a state that then induces the behavioural response. Whereas the two mentioned pathways may both result in elevated positive emotional states that are likely to benefit the playmates in multiple ways (for benefits of play, see [4,5]) it is difficult to determine which is the underlying path for the various forms of matching in animal play. What we know with more certainty is that any involved emotion state changes are likely to be minimal if the studied animals are already playfully interacting, i.e. in the same social–emotional context.

To systematically test for positive emotional contagion, where an emotional state spreads across individuals, it is important to have subjects socially and emotionally disconnected and, preferably, to examine them beyond a dyadic level [131,132]. An interesting study by Schwing and colleagues [75] on keas, a playful parrot species, demonstrated that played-back recordings of play vocalizations induce play behaviours in conspecifics previously involved in other behaviours. This study supports the notion that positive emotional contagion might not be a human-unique phenomenon. Interestingly, similar playback approaches carried out with chimpanzee laughter recordings did not show a comparable outcome in their conspecifics [133,134]. Unlike humans [135], chimpanzees do not seem to produce laughter merely based on hearing such vocalizations.

Mimicking and other types of behavioural matching within the context of play are also likely to be important for socially learning and practising a wide range of behaviours in humans and nonhuman animals [4,5,136]. In support of this notion, there is evidence that animals match the exact variant of the same expression of their playmates [58,112] and that the matching of play expressions may differ in form and function between social groups [86,137,138]. This brings us back to the Power Asymmetry Hypothesis [59], which could be extended to colony differences. Colonies may differ in the degree in which they show tolerance and aggression [139,140] and it seems reasonable to argue that clearer forms of communication may be essential when there is more risk of getting hurt (see [59]). Furthermore, the absence of the exposed upper teeth in the laughing chimpanzees at Burgers' Zoo, mentioned by Jan van Hooff in his pioneering work [41], and its presence in other chimpanzee colonies (see [26]) might indicate group differences regarding this facial feature. Exact matching of facial variants could help explain such potential differences. However, this topic requires further research.

Interestingly, there is some indication that the upper-teeth exposure develops at a later stage in immature primates [57,105], so that its occurrence throughout the developmental trajectory could depend on the exact matching mechanism and the social environment. More research is, however, needed on this topic. In a recent psychoacoustic study, Kret *et al.* [141] played back human infant laughter to adult participants, who were asked to determine the airflow direction from the recordings. The researchers found that the infants produced laughter increasingly as an egressive vocalization (i.e. a vocalization that is produced during the exhalation phase only; see [22,142]) over time and that this acoustic trait was perceived to be more positive by the adult listeners [141]. Such developmental findings could indicate that human infants already adjust laughter to their acoustic environment via social feedback.

## 4. Conclusion

All in all, empirical findings on primates, carnivores and other animals reveal a complexity in the facial and vocal expressions of social play in both form and function, in line with the Complexity and Continuity Hypothesis [33]. These positively grounded expressions seem to have multiple functions across species—among others, to promote social bonding. Despite such shared complexity of homologous and homoplastic playful expressions that most likely already existed throughout the main part of mammal evolution, closer toward the hominin lineage, they must have been produced more flexibly free from behavioural contexts to become more powerful tools of everyday social interactions in humans.

## Data Availability

The data are available in the manuscript (table 1).
